# Dicephalus Parapagus Conjoined Twins Diagnosed by First-Trimester Ultrasound

**DOI:** 10.1155/2016/8565193

**Published:** 2016-06-05

**Authors:** Keiko Watanabe, Masanori Ono, Mayu Shirahashi, Toshiyuki Ikeda, Kazumi Yakubo

**Affiliations:** Department of Obstetrics and Gynecology, Saitama City Hospital, Midori, Saitama 336-8522, Japan

## Abstract

Conjoined twins are a rare phenomenon, occurring in 1% of monochorionic twin gestation, with an incidence of 1 : 50 000 to 1 : 100 000. Many conjoined twins have abnormalities incompatible with life, so early prenatal diagnosis is very important for optimal management of both pregnancy and delivery. We report a case of dicephalus parapagus conjoined twins, sharing a single heart, diagnosed at 12 weeks' gestation. With early ultrasound diagnosis, we were able to provide appropriate and timely prenatal counseling to the family.

## 1. Introduction

Conjoined twins are always monochorionic, with either fused or partially fused anatomy [1]. The incidence varies from 1 : 50 000 to 1 : 100 000, with the condition occurring in 1% of monochorionic twins [2–4]. The fission theory proposes that conjoined twins represent a fertilized ovum that divides incompletely [5]. Per the theory of secondary fusion, the condition results from 2 originally distinct monovular embryos [6, 7]. The prognosis is not good, with 60% of conjoined-twin gestation resulting in miscarriage or stillbirth [2]. Many conjoined twins have abnormalities incompatible with life [2, 8], and the condition is also associated with various maternal complications [2]. Accurate prenatal imaging is crucial in diagnosing this rare entity. We present a patient in whom the early prenatal diagnosis of conjoined twins allowed us to provide appropriate, timely antenatal counseling.

## 2. Case Report

**Figure 1:**
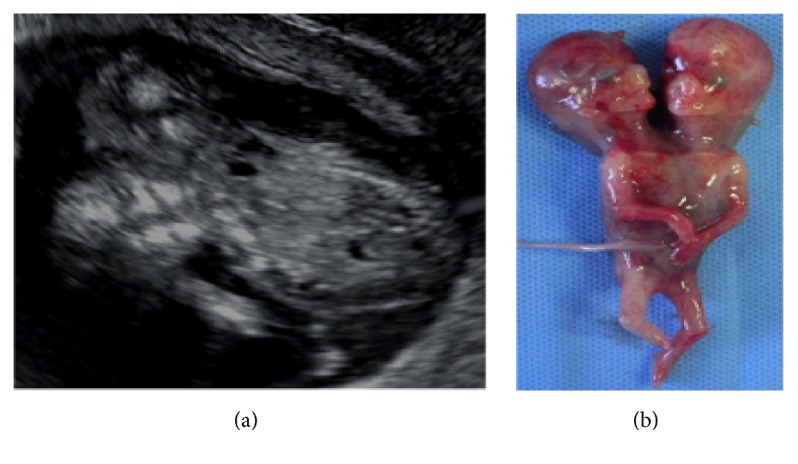
(a) Ultrasound showing twin gestation with fusion of the thorax and abdomen; (b) ex vivo photograph showing dicephalus parapagus conjoined twins.

A 33-year-old primigravid woman was referred to our hospital at 12 weeks' gestation with a suspected monochorionic-monoamniotic twin pregnancy. She had no significant medical or family history and no exposure to medications, radiation, or infection. At her first visit, we confirmed the diagnosis of monochorionic-monoamniotic twin pregnancy, with ultrasound revealing only a single trunk with 2 heads in close apposition, implying the possibility of conjoined twins (Figure 1(a)). Five days later, we confirmed that conjoined twins were present. The fetuses shared 2 heads, 1 trunk, a single heart, 2 upper limbs, and 2 lower limbs; the final diagnosis was dicephalic parapagus conjoined twins (Figure 1(b)). The couple was informed of these findings and underwent prenatal counseling at 13 weeks' gestation.

## 3. Discussion

Conjoined twins are a rare occurrence, with a female predominance as high as 3 : 1 [2]. No association with maternal age, race, parity, or heredity has been observed. Ultrasound is very useful for diagnosis [9]; various clues that may be observed include unusually close fetal apposition, spinal extension, and a single heart. Once the diagnosis of conjoined twins is made, it is necessary to characterize the type and severity of the abnormality in order to estimate the chances for the infants' survival after delivery.

Conjoined twins are classified according to the most prominent part of interconnection [10]. There are many possible sites of fusion, resulting in several possible diagnoses: cephalopagus, thoracopagus, omphalopagus, ischiopagus, parapagus, craniopagus, rachipagus, and pygopagus [11]. Our patient's twins were dicephalus parapagus, sharing a conjoined pelvis, a single symphysis pubis, and a single trunk with 2 heads. In some cases, surgical separation of conjoined twins may be successful. Unfortunately, our patient's twins shared a single heart, making surgical separation incompatible with life. We were able to inform our patient and her partner of this ominous prognosis.

Conjoined twins are a rare occurrence, but any monochorionic-monoamniotic gestation must be carefully evaluated for any evidence of conjoined bodies. Once diagnosed, conjoined twins must be classified by type in order to determine the prognosis. Early prenatal diagnosis can allow clinicians to provide appropriate and timely counseling.

## References

[1] Mutchinick O. M., Luna-Muñoz L., Amar E. (2011). Conjoined twins: a worldwide collaborative epidemiological study of the International Clearinghouse for Birth Defects Surveillance and Research. *American Journal of Medical Genetics Part C: Seminars in Medical Genetics*.

[2] Spitz L. (2005). Conjoined twins. *Prenatal Diagnosis*.

[3] Chen C.-P., Hsu C.-Y., Su J.-W. (2011). Conjoined twins detected in the first trimester: a review. *Taiwanese Journal of Obstetrics and Gynecology*.

[4] Boyle B., McConkey R., Garne E. (2013). Trends in the prevalence, risk and pregnancy outcome of multiple births with congenital anomaly: a registry-based study in 14 European countries 1984–2007. *BJOG: An International Journal of Obstetrics and Gynaecology*.

[5] Kaufman M. H. (2004). The embryology of conjoined twins. *Child's Nervous System*.

[6] Spencer R. (2000). Theoretical and analytical embryology of conjoined twins: part II: adjustments to union. *Clinical Anatomy*.

[7] DeStephano C. C., Meena M., Brown D. L., Davies N. P., Brost B. C. (2010). Sonographic diagnosis of conjoined diamniotic monochorionic twins. *American Journal of Obstetrics and Gynecology*.

[8] Yang P. Y., Wu C. H., Yeh G. P., Hsieh C. T. (2015). Prenatal diagnosis of parapagus diprosopus dibrachius dipus twins with spina bifida in the first trimester using two- and three-dimensional ultrasound. *Taiwanese Journal of Obstetrics and Gynecology*.

[9] McHugh K., Kiely E. M., Spitz L. (2006). Imaging of conjoined twins. *Pediatric Radiology*.

[10] McMahon C. J., Spencer R. (2006). Congenital heart defects in conjoined twins: outcome after surgical separation of thoracopagus. *Pediatric cardiology*.

[11] Baken L., Rousian M., Kompanje E. J. O. (2013). Diagnostic techniques and criteria for first-trimester conjoined twin documentation: a review of the literature illustrated by three recent cases. *Obstetrical and Gynecological Survey*.

